# Top-Down Control of Inhibitory Granule Cells in the Main Olfactory Bulb Reshapes Neural Dynamics Giving Rise to a Diversity of Computations

**DOI:** 10.3389/fncom.2020.00059

**Published:** 2020-07-13

**Authors:** Zhen Chen, Krishnan Padmanabhan

**Affiliations:** ^1^Department of Brain and Cognitive Sciences, University of Rochester, Rochester, NY, United States; ^2^Department of Neuroscience, University of Rochester School of Medicine and Dentistry, Rochester, NY, United States

**Keywords:** dynamical system, olfactory bulb, oscillations, pattern separation, synchrony, bifurcation, feedback, top-down

## Abstract

Growing evidence shows that top-down projections from excitatory neurons in piriform cortex selectively synapse onto local inhibitory granule cells in the main olfactory bulb, effectively gating their own inputs by controlling inhibition. An open question in olfaction is the role this feedback plays in shaping the dynamics of local circuits, and the resultant computational benefits it provides. Using rate models of neuronal firing in a network consisting of excitatory mitral and tufted cells, inhibitory granule cells and top-down piriform cortical neurons, we found that changes in the weight of feedback to inhibitory neurons generated diverse network dynamics and complex transitions between these dynamics. Changes in the weight of top-down feedback supported a number of computations, including both pattern separation and oscillatory synchrony. Additionally, the network could generate gamma oscillations though a mechanism we termed **T**op-down control of **I**nhibitory **N**euron **G**amma (TING). Collectively, these functions arose from a codimension-2 bifurcation in the dynamical system. Our results highlight a key role for this top-down feedback, gating inhibition to facilitate often diametrically different computations.

## Introduction

Growing evidence suggests that top-down centrifugal feedback from higher cortical areas specifically target inhibitory interneurons in primary sensory regions. In the olfactory system, axons from excitatory neurons in the piriform cortex (PCx) synapse onto the inhibitory granule cells in olfactory bulb (OB), whereby they can modulate the function of the mitral/tufted cells (M/T), the principal relays of olfactory information from the bulb to the brain (Shipley and Adamek, 1984; Boyd et al., 2012; Markopoulos et al., 2012; Oswald and Urban, 2012b; Padmanabhan et al., 2016, 2019). This circuit motif results in piriform cortical neurons receiving input from only excitatory M/T cells but exerting influence on the local circuit dynamics in the OB via inhibitory populations. Consequently, the information relayed to piriform cortical neurons comes from M/T cells, but feedback intervenes in network dynamics through the local inhibitory granule cells. Although this network motif constitutes major feature of the olfactory system, the computational role of this top-down control of inhibition remains largely unknown.

A number of studies have previously explored the dynamics of M/T cells and granule cells in OB as a two-population network of excitatory (E) and inhibitory (I) neurons (Cleland and Linster, 2005; Brea et al., 2009; Kay et al., 2009; Li and Cleland, 2017) in parallel with a broader literature on excitatory-inhibitory (E-I) networks (Wilson and Cowan, 1972; Ermentrout and Kopell, 1990; Tsodyks et al., 1997; Tiesinga and Sejnowski, 2009; Ledoux and Brunel, 2011; Franci et al., 2018). The studies in olfaction have revealed not only the mechanisms by which these dynamics emerge, but also how changes in the oscillatory power (Nusser et al., 2001) result in alterations in behavior, including in odor discrimination tasks (Abraham et al., 2010). The recent evidence that cortical feedback directly synapses onto inhibitory interneurons (Boyd et al., 2012; Markopoulos et al., 2012) suggests that the local dynamics of excitatory and inhibitory neurons in OB can be gated by centrifugal projections from olfactory cortex. How the local E-I network's activity in the bulb is changed by centrifugal input, what these changes mean more broadly for neural dynamics in the early olfactory system, and the role of these dynamics play in neural computation remains an open question.

To address this, we built a three-node network model consisting of an excitatory population of mitral/tufted cells (M), an inhibitory population of granule cells (G) and a top-down population of pyramidal/semilunar cells (P) in PCx, and studied how firing rate dynamics were influenced by top-down weights onto inhibition. Changing the weight of the top-down connections to local inhibitory neurons reshaped the dynamics of the local E-I circuit in a way that enhanced sensory discrimination as well as generated oscillatory synchrony including entraining gamma oscillations in the local circuit [**T**op-down control of **I**nhibitory **N**euron **G**amma, (TING)]. Finally, the mechanism underlying the dynamics, as well as the functional roles played by top-down control of inhibition occurred via a codimension-2 bifurcation in the dynamical system. By gating the weight of connections from piriform cortex to the inhibitory neurons in the bulb, a number of seemingly disparate computations could be supported by a single circuit, providing an additional framework for the diversity of inhibitory interneuron function in the olfactory bulb.

## Materials and Methods

### Network Model

**Figure 1:**
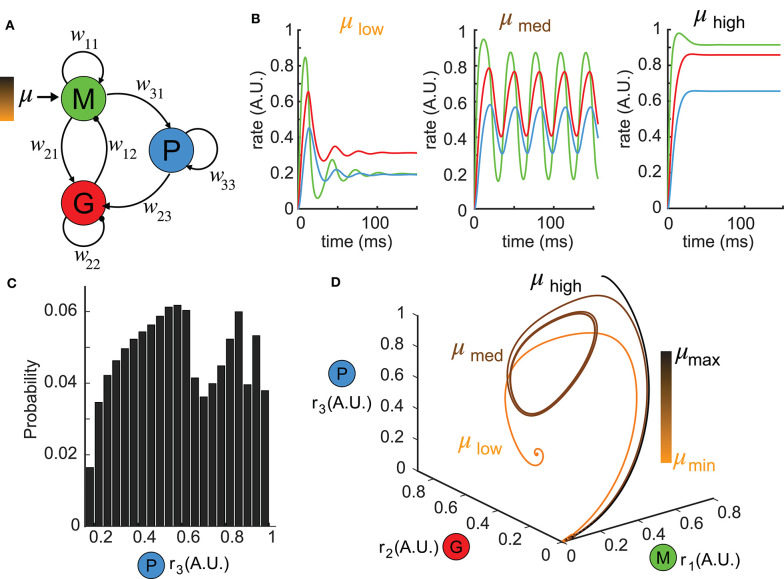
Reduced network model exhibiting complex dynamics. **(A)** A schematic diagram illustrating the topology of the reduced network model. Each node denotes a neuron population and the connection weights are defined by *w*_*ij*_ (excitatory: arrows; inhibitory: circles). **(B)** Network responses [color-coded firing rates match population nodes in **(A)**] to different levels of stimulus strength (left: μ_*low*_ = 0.1, middle: μ_*med*_ = 1.5, right: μ_*high*_ = 3.0) with the other parameters fixed. **(C)** A distribution of all possible steady states *r*_3_ over a wide range of parameter choices: μ ∈ [0, 5], *w*_31_ ∈ [0, 5], *w*_23_ ∈ [0, 30] shows the diversity of responses the network can generate. **(D)** Trajectories of the firing rate responses plotted in **(B)** are visualized in the phase space spanned by (*r*_1_, *r*_2_, *r*_3_). The color bar indicates stimulus strengths for three representative levels as in **(B)**.

The network model was composed of three nodes, the local excitatory population, corresponding to mitral and tufted cells (M) and inhibitory population corresponding to granule cells (G) which were reciprocally coupled, and a top-down population corresponding to the principal neurons in piriform cortex (P) that received input from the local M population and projected back to the inhibitory G population (Figure 1A). In the model, *r*_*i*_ (*t*), *i* = 1, 2, 3 represented the firing rates of the three neuron populations, respectively, whose dynamics were determined by Wilson-Cowan equations (Wilson and Cowan, 1972) as follows:

(1) $$\{\begin{array}{c} \tau_{1}\dot{r_{1}}=-r_{1}+S\left(w_{11}r_{1}+w_{12}r_{2}+\mu \right)\\ \tau_{2}\dot{r_{2}}=-r_{2}+S\left(w_{21}r_{1}+w_{22}r_{2}+w_{23}r_{3}\right)\\ \tau_{3}\dot{r_{3}}=-r_{3}+tanh\left(w_{31}r_{1}+w_{33}r_{3}\right) \end{array}$$

where *S* is the sigmoid function:

(2) $$\begin{array}{l} S\left(x\right)=\frac{1}{1+e^{-x}} \end{array}$$

which described the non-linear relationship between the mean synaptic input and average firing rate (normalized to a range between 0 and 1). The parameter τ_*i*_, *i* = 1, 2, 3 was the time constant for each population, characterizing how quickly the dynamics of each population evolved. The mitral/tufted cell population (M) received an external stimulus μ, that represented the only external input to the system. The connection weight from population *j* to population *i* was denoted by *w*_*ij*_, *i, j* = 1, 2, 3, among which *w*_11_, *w*_21_, *w*_31_, *w*_23_, *w*_33_ > 0 and *w*_12_, *w*_22_ < 0. The connection weight *w*_*ij*_, *i, j* = 1, 2, 3 represented the average synaptic input received by the neuron population i from the population j. Throughout this paper, we set the parameters as follows: *w*_11_ = 8.7, *w*_12_ = −10, *w*_21_ = 7.0, *w*_22_ = −13, *w*_31_ = 1.5, *w*_33_ = 0.5. The parameters in the model were chosen based on previous studies of Wilson-Cowan rate model (Ermentrout and Kopell, 1990; Ledoux and Brunel, 2011; Veltz and Sejnowski, 2015), and their relative values were adjusted according to experimentally recorded excitatory and inhibitory postsynaptic inputs of M/T and granule cells in OB (Urban and Sakmann, 2002; Egger et al., 2005; Kapoor and Urban, 2006). For instance, the value of recurrent excitation (*w*_11_) was determined by mapping recorded excitatory post-synaptic potentials (EPSP) in M/T cells and the weight of inhibition from granule cells (*w*_12_) was determined using similar mappings of whole cell recordings of inhibitory post-synaptic potentials (IPSP) in M/T cells (Urban and Sakmann, 2002). The weights between populations were determined by integrating synaptic potentials with known connectivity densities using trans-synaptic viral tracers that allowed for estimates of the number of pre-synaptic cells for each population (Willhite et al., 2006; Miyamichi et al., 2011; Padmanabhan et al., 2016) to yield the relative weights for instance, |*w*_12_| > |*w*_21_|. Estimates of synaptic weights for the projections to piriform cortex and the feedback connections were estimated from Franks and Isaacson (2006), Suzuki and Bekkers (2011), and Boyd et al. (2012). Time constants, for example the time constant τ_2_ for granule cells, were derived from data using both calcium imaging and whole cell recordings across the types of neurons that constituted the populations in our model (Franks and Isaacson, 2006; Kapoor and Urban, 2006; Suzuki and Bekkers, 2011). As there was no inhibitory synaptic input into the piriform pyramidal/semilunar cell population (P), the combination *w*_31_*r*_1_ + *w*_33_*r*_3_ was non-negative due to *w*_31_ > 0 and *w*_33_ > 0. Sigmoid function (2) for the third equation of system (1) would mean that the lowest *r*_3_ value that an equilibrium could reach would be 0.5. In this framework, even without an input, at least half of the pyramidal/semilunar cells would keep firing. Thus, we used the hyperbolic tangent function *tanh* (*x*) in order ensure that piriform cortical cell firing rates were low in the absence of odors (Stettler and Axel, 2009; Davison and Ehlers, 2011), and to exploit the entire range [0, 1] of *r*_3_ (Figure 1C). However, it should be noted that such choice of non-linearity did not affect our findings since all bifurcations supporting the computations of the network we identified were found in the system using the sigmoid function (2).

### Definition of Period of a Limit Cycle

From the perspective of dynamical system, a limit cycle in the phase space corresponds to the oscillation of firing rates in the temporal space. Since the time constants in our model had units of millisecond, the frequency of oscillations was defined as 1, 000/*T*, with *T* denoting the period of limit cycles, which was defined as follows: if *r*_*i*_ (*t*), *i* = 1, 2, 3 denote the firing rate of neural population in the model, then a limit cycle satisfies the periodicity:

(3) $$\begin{array}{l} r_{i}\left(t\right)=r_{i}\left(t+T\right),\;i=1,2,3 \end{array}$$

for some *T* > 0 and all *t*∈ ℝ. The minimal *T* for which the above equality holds is the period of the limit cycle.

### Metric Definition

As representations of the network (ω-limit sets) could take on different forms, an equilibrium or a limit cycle, we defined two quantitative metrics: $d^{E}\left(\Omega_{1},\Omega_{2}\right)$ and $d^{S}\left(r_{3}{|}_{\Omega_{1}},r_{3}{|}_{\Omega_{2}}\right)$ which served to measure the distance between different types of ω-limit sets in responses to any given stimulus pair (μ_1_, μ_2_) where μ_2_ = μ_1_ + Δ. $d^{E}\left(\Omega_{1},\Omega_{2}\right)$ denoted the average Euclidean distance between Ω_1_ and Ω_2_ in the three-dimensional (3D) phase space of firing rates (*r*_1_, *r*_2_, *r*_3_), and the spectrum distance $d^{S}\left(r_{3}{|}_{\Omega_{1}},r_{3}{|}_{\Omega_{2}}\right)$ was a sum of the squared differences between both direct components (DC) and alternating components (AC) in the amplitude-frequency domain of the Fourier transforms to the signals *r*_3_ (*t*) associated with the two stimuli.

The Euclidean distance was defined as follows: supposing that Ω_1_ and Ω_2_ are two ω-limit sets composed of *N*_1_ and *N*_2_ discrete points in three-dimensional phase space (*r*_1_, *r*_2_, *r*_3_), respectively, denoted as {***α***_1_, ***α***_2_, …, ***α***_*N*_1__} and {***β***_1_, ***β***_2_, …, ***β***_*N*_2__} where ***α***_*i*_ and ***β***_*j*_ are three-dimensional vectors, then

(4) $$d^{E}=\langle d\left(\alpha_{i},\;\beta_{j}\right)\rangle ,\;i=1,\ldots ,\;N_{1},\;j=1,\ldots ,\;N_{2}$$

Where 〈·〉 denotes the average and $d\left(x,y\right)=\sqrt{{\left(x-y\right)}^{T}\left(x-y\right)}$ which is the standard distance between any two points in the three-dimensional Euclidean space. Note that in the case of equilibria, the number of discrete points in the ω-limit set was one: *N* = 1, and in the case of a limit cycle, we set *N* = *T*/*dt*, where *T* was the period and *dt* was time bin for numerical integration.

The Euclidean distance *d*^*E*^ worked well in measuring the distance between two equilibria in the phase space, but when one ω-limit set was a limit cycle, the averaging operation in the definition made it only a coarse and lagged estimate of the separation for equilibrium vs. limit cycle and limit cycle vs. limit cycle. In particular, the activity of the P population should be decodable with respect to the stimulus information, something that was problematic for when using *d*^*E*^. Therefore, we defined the spectrum distance *d*^*S*^ to address the question of distances between representations that were sensitive to those representations being oscillations. To calculate the spectrum distance *d*^*S*^ between two ω-limit sets Ω_1_ and Ω_2_, only the sequence of their *r*_3_ component was decomposed by Fourier transform which converted the firing rate signal in the temporal domain into a representation in the frequency domain.

The single-sided amplitude spectrum for the Fourier transform of the firing rate signal *r*_3_ (*t*), was used to obtain peaks around frequency values. For an equilibrium corresponding to constant firing rate *r*_3_ = *A*, there existed only one peak around zero frequency with its amplitude proportional to *A*, since the Fourier transform of a constant function is a delta-function. We referred this component as the direct component (DC) of the signal. For the case of a limit cycle, in addition to one peak around zero frequency, there existed another peak around frequency 1, 000/*T* where *T* denoted the period of the limit cycle. We referred this additional peak of a limit cycle as the alternating component (AC). Thus, the spectrum distance *d*^*S*^ was a sum of the differences between both direct components (DC) of two limit sets and alternating components (AC) of two limit sets, which was formalized as follows: supposing that *D*_1_ and *D*_2_ were the amplitudes of the peaks at zero frequency for two ω-limit sets Ω_1_ and Ω_2_, and **a**_*i*_ **=** (*f*_*i*_, *A*_*i*_)**,** *i* = 1, 2 denoted the corresponding alternating components of Ω_*i*_, where *f*_*i*_ was the non-zero frequency and *A*_*i*_ was the amplitude of the peak around *f*_*i*_, then we have

(5) $$\begin{array}{l} d^{S}=|D_{1}-D_{2}|+d\left(a_{1},a_{2}\right) \end{array}$$

Note that we set ***a***_*i*_ **=** (0, 0) if the ω-limit sets Ω_*i*_ was an equilibrium. Thus, when the two limit sets were both equilibria, *d*^*S*^ only contained the first term measuring the difference between the direct components. In this case, *d*^*S*^ was only a linear projection (up to a constant factor) of the Euclidean distance *d*^*E*^.

**Figure 2:**
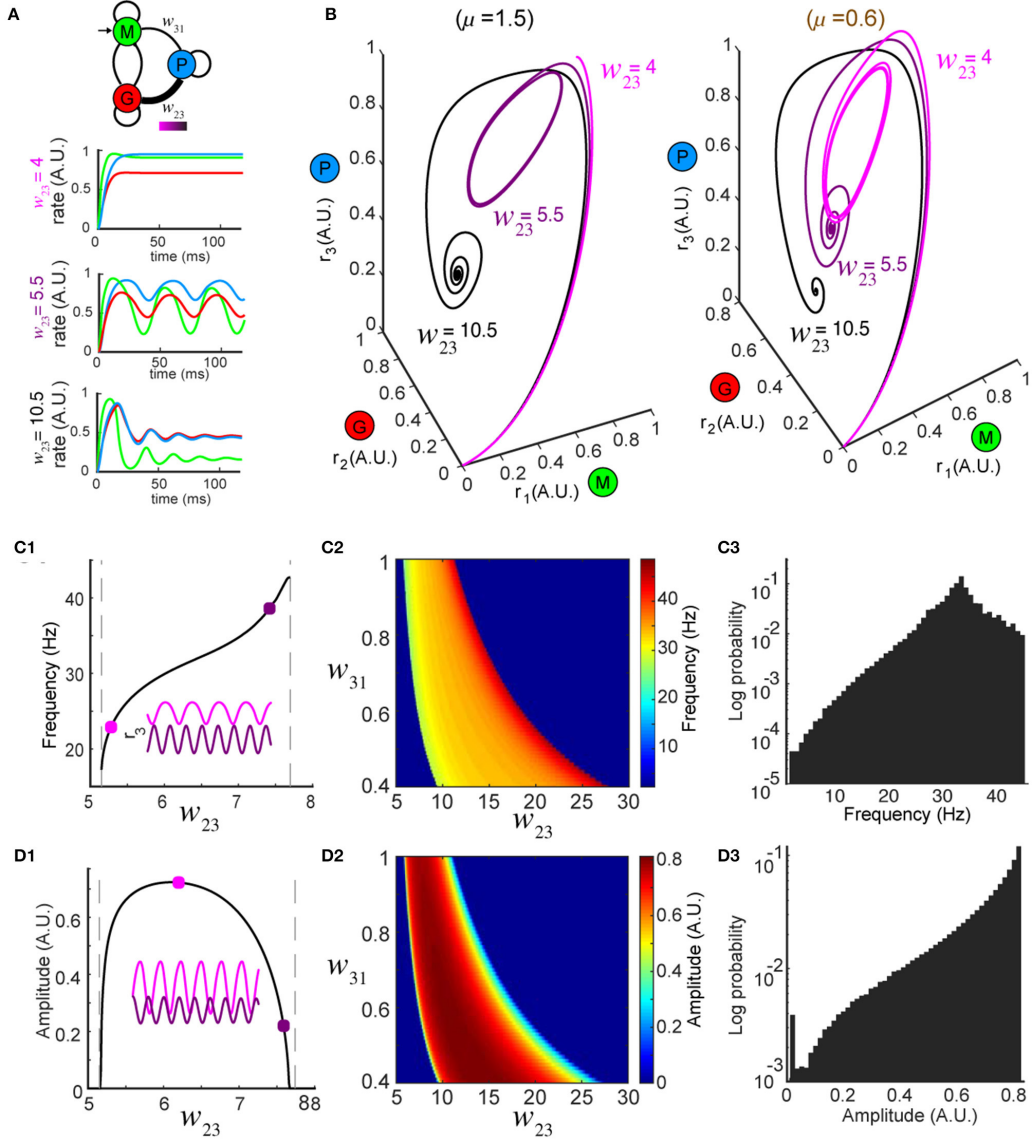
Network dynamics are controlled by top-down modulation. **(A)** Top: changing top-down input *w*_23_ reshapes network activity to the same stimulus (color bar indicates values of *w*_23_); bottom: firing rates at three representative values of *w*_23_ while the stimulus is held constant (μ = 1.5). **(B)** Trajectories in the phase space for the same *w*_23_ as in **(A)**; left: oscillations occur around *w*_23_ = 5.5 for μ = 1.5 (same as **A**); right: oscillations occur around *w*_23_ = 10.5 for a different stimulus μ = 0.6, revealing that the dynamics are diverse across different combinations of stimuli and top-down input. **(C)** Modulation of oscillation frequency by top-down input (for μ = 1.5). **(C1)** Dependence of frequency on top-down input *w*_23_. Inset: time series of *r*_3_ (*t*) for two example values of *w*_23_ (squares). **(C2)** Frequency modulation by top-down input occurred over a range of feedforward drive *w*_31_. **(C3)** A distribution of oscillation frequencies that can be generated by the network for all possible combinations of *w*_23_ ∈ [0, 30] and μ ∈ [0, 5]. **(D)** Similar to **(C)** but for oscillation amplitudes.

**Figure 3:**
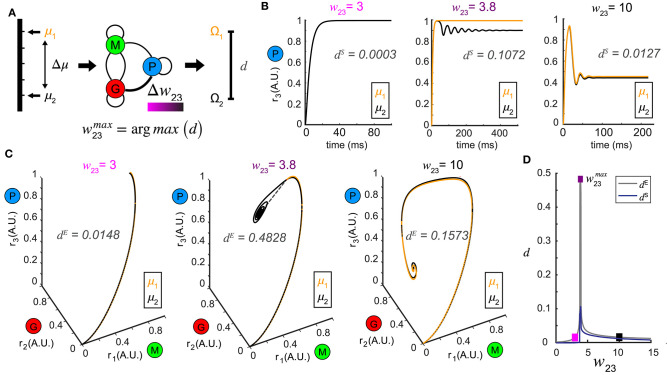
Pattern separation via top-down control. **(A)** A schematic diagram illustrating the separation maximization between the response patterns to a pair of stimuli (μ_1_ and μ_2_) by changing top-down input *w*_23_ (color bar indicates values of *w*_23_). **(B)** Time series of *r*_3_ (*t*) in response to μ_1_ = 1.0 and μ_2_ = 1.1 at three representative values of *w*_23_ with the spectrum distance *d*^*S*^ indicated. The two responses *r*_3_ (*t*) are close at some top-down inputs (left: *w*_23_ = 3; right: *w*_23_ = 10), but pushed apart at other top-down input (middle: *w*_23_ = 3.8). **(C)** Phase trajectories and network representations of two stimuli from which the Euclidean distance *d*^*E*^ is calculated. From left to right the top-down input *w*_23_ correspond to those in **(B)** for the same stimulus pair (μ_1_ = 1.0 and μ_2_ = 1.1). **(D)** Non-monotonic dependence of both *d*^*E*^ and *d*^*S*^ on top-down input *w*_23_, with maximum achieved at *w*_23_ = 3.8. The squares are color coded as in **(B,C)**.

**Figure 4:**
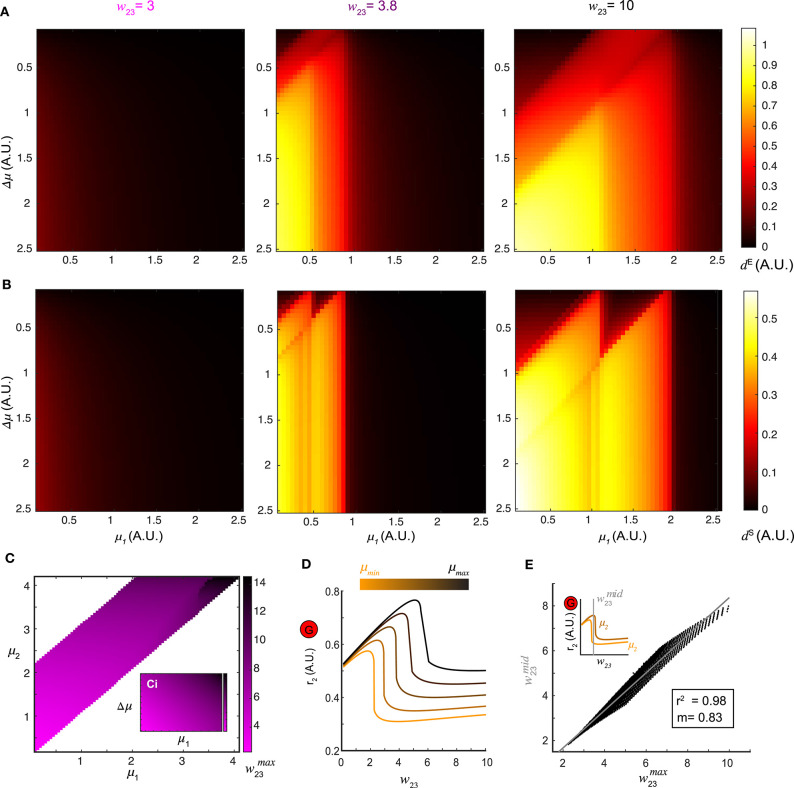
Optimal top-down input for pattern separation. **(A)** Landscapes of the Euclidean distance *d*^*E*^ over all stimulus pairs at three representative values of top-down input. **(B)** Landscapes of the spectrum distance *d*^*S*^ unfolds as in **(A)**. **(C)** The matrix $w_{23}^{\max}$ which maximizes *d* between network representations in response to all combinations of stimuli (μ_1_ and μ_2_) presented to the network. Inset: the same matrix of $w_{23}^{\max}$ organized by one stimulus μ_1_ vs. stimulus difference Δμ. **(D)** Dependence of the stationary firing rate of inhibitory population *r*_2_ on feedback *w*_23_ at different levels of input strength (indicated by color bar). **(E)** The correlation between $w_{23}^{\max}$ obtained from **(C)** for all pairs of stimuli μ_1_ and μ_2_ and the $w_{23}^{mid}$ corresponding to the mid-point of the two inhibitory firing rate maxima associated with the same pair of stimuli (upper left inset) reveals that top-down input optimizes pattern separation by gating the G-M inhibition as well as recurrent G inhibition. The gray line denotes the utility line. Lower right inset shows the correlation coefficient and the slope of the linear regression.

When both Ω_1_ and Ω_2_ were equilibria, the spectrum distance *d*^*S*^ was a projection of the Euclidean distance *d*^*E*^ onto the *r*_3_ axis. The spectrum distance was however more sensitive to bifurcations when one of the two ω-limit sets Ω_1_ and Ω_2_ transitioned into a limit cycle as the frequency of the limit cycle started from non-zero values at the onset of a bifurcation (Figure 2C1), causing discontinuous jumps in the spectrum distance. However, for the purpose of pattern separation, the two metrics did not give qualitatively different results when assessing the distances due to changes the feedback weight *w*_23_ (**Figure 4**). Additionally, we found that the non-monotonic dependence of distance in both *d*^*E*^ and *d*^*S*^ on the feedback weight ($w_{23}^{\max}$) persisted, and that an optimal value for any given pair of stimuli could be found (Figure 3D).

### Bifurcation Analysis

We denoted the dynamical system of Equation (1) as a parameterized form

(6) $$\dot{r}=f\left(r,\Theta \right)$$

where ***r*** ∈ ℝ^3^ was the vector of firing rates and Θ∈ ℝ^*p*^ was the vector of parameters. The vector field $f\;=\;{\left(f_{1},f_{2},f_{3}\right)}^{T}$ was a smooth function on some open set of ℝ^3^ × ℝ^*p*^. The dimensionality of Θ could be up to eight dimensions maximally to include all connection weights *w*_*ij*_ and the external stimulus μ. However, since we were only interested in the top-down control, Θ was restricted to be two dimensions (*p* = 2) including the top-down weight *w*_23_ and the external stimulus μ. All the other parameters were fixed as constants determined based on previous experimental work (Whittington et al., 2000). As the system Equation (6) had an equilibrium at (***r***, Θ) **=** (***r***_0_**,** Θ_0_), i.e.,

(7) $$\begin{array}{l} f\left(r_{0},\;\Theta_{0}\right)=0 \end{array}$$

the stability of this equilibrium could be determined from the linearized vector field of Equation (6) given by

(8) $$\dot{\xi }=D_{r}f\left(r_{0},\Theta_{0}\right)\xi ,\xi \in \;{\mathbb{R}}^{3}$$

where $D_{r}f≜\frac{\partial f}{\partial r}$ was the Jacobian matrix of the vector field **f**.

If none of the eigenvalues of *D*_***r***_***f*** (***r***_0_, Θ_0_) lied on the imaginary axis (i.e., the equilibrium was *hyperbolic*), the local stability of (***r***_0_, Θ_0_) in the non-linear system (6) could be determined by the linear system (8). The equilibrium was stable if all eigenvalues of *D*_***r***_***f*** (***r***_0_, Θ_0_) had negative real parts. In the case of a hyperbolic equilibrium, varying slightly the parameter Θ would not change the stability as taking Equation (7) and the invertibility of *D*_***r***_***f*** (***r***_0_, Θ_0_), there existed a unique smooth function ***h*** : ℝ^*p*^ → ℝ^3^ such that

(9) $$\begin{array}{l} h\left(\Theta_{0}\right)=r_{0}\text{ and }f\left(h\left(\Theta \right),\Theta \right)=0 \end{array}$$

for Θ sufficiently close to Θ_0_. By continuity of the eigenvalues with respect to parameters, *D*_***r***_***f*** (***h*** (Θ), Θ) had no eigenvalue on the imaginary axis for Θ sufficiently close to Θ_0_. Therefore, the hyperbolicity of the equilibrium persisted and its stability type remained unchanged for in close vicinity of Θ_0_. By contrast, when some of the eigenvalues of *D*_***r***_***f*** (***r***_0_, Θ_0_) lied on the imaginary axis, for example, a zero eigenvalue or a pair of purely imaginary eigenvalues, new topologically different dynamical behaviors occurred by a small change in Θ. Equilibria could be created or annihilated, and periodic dynamics could emerge.

The parameterized system (6) thus underwent a bifurcation at (***r***_0_, Θ_0_) if the Jacobian matrix *D*_***r***_***f*** (***r***_0_, Θ_0_) has an eigenvalue of zero real part. In our model, a saddle-node bifurcation (SN) occurred when *D*_***r***_***f*** (***r***_0_, Θ_0_) had a single zero eigenvalue (in addition to some non-degenerate conditions), and a Hopf bifurcation (H) occurred when *D*_***r***_***f*** (***r***_0_, Θ_0_) had a pair of purely imaginary eigenvalues. The bifurcation point was found numerically by XPPAUT or the Matlab toolbox MATCONT.

The number of parameters that must be varied simultaneously to evoke a bifurcation is defined as the *codimension* of this bifurcation (Guckenheimer and Holmes, 2013; Kuznetsov, 2013). Considering the infinite-dimensional space H of all vector fields defined on the *n*-dimensional Euclidean space ℝ^*n*^, a vector field **f**_0_ undergoing a bifurcation, for example, a Hopf bifurcation, corresponds to a point in the space H. All nearby vector fields with the same singularity as ***f***_0_ (i.e., vector fields that are orbitally topologically equivalent to ***f***_0_) form a submanifold L of co-dimension *k*, which is an equivalence class of the singular vector field ***f***_0_. Therefore, within the space H it requires another submanifold $\tilde{L}$ of at least *k* dimensions to intersect transversely with L at point ***f***_0_, such that the singularity of ***f***_0_ persists under small perturbations of the vector field. The submanifold $\tilde{L}$ was obtained through a parametrized family of vector fields involving at least *k* parameters. The least number *k* is then defined as the codimension of ***f***_0_. The parametrized vector field ***f*** (***r***, Θ) in Equation (6) can be thought of as one realization of the submanifold $\tilde{L}$ which passes through the vector field ***f***_0_ ≜ ***f*** (***r***_0_, Θ_0_) undergoing a bifurcation with the two parameters corresponding to the top-down weight *w*_23_ and the external stimulus μ.

### Simulating Sniffing With a Periodically Driven Non-autonomous System

Olfactory sensation is an active process, with sensory stimuli being sampled by sniffing on the time scale of 215Hz in animal models (Carey and Wachowiak, 2011; Wachowiak, 2011). To simulate sniffing, a vector field for the dynamical system that depended explicitly on time and was also periodic with fixed period *T* = 2π / ω > 0, i.e.,

(10) $$\begin{array}{l} \dot{r}\;\;=\;f\left(r,\;t\right)\;and\;f\left(r,\;t+T\right)=f\left(r,\;t\right) \end{array}$$

could be rewritten in the form of an autonomous system by defining the function

(11) $$\begin{array}{l} \varphi \left(t\right)\;=\;\omega t,\;mod\;2\pi \end{array}$$

such that using Equation (11), Equation (10) became

(12) $$\begin{array}{l} \begin{array}{c} \dot{r}\;=\;f\left(r,\;\varphi \right)\\ \dot{\varphi }=\omega \end{array}\;\left(r,\;\varphi \right)\in {\mathbb{R}}^{3}\times S^{1} \end{array}$$

where *S*^1^ denoted a circle. To construct the Poincaré map, we defined a cross-section of Equation (12) by

(13) $$\begin{array}{l} \Sigma_{0}^{\bar{\varphi }}=\left\{\left(r,\;\varphi \right)\in {\mathbb{R}}^{3}\times S^{1}\;|\varphi ={\bar{\varphi }}_{0}\in \left(0,\;2\pi \right]\;\right\} \end{array}$$

such that a fixed point of the Poincaré map $P_{{\bar{\varphi }}_{0}}:\Sigma^{{\bar{\varphi }}_{0}}\to \Sigma^{{\bar{\varphi }}_{0}}$ corresponded to a limit cycle of the extended system Equation (12), and a limit cycle of $P_{{\bar{\varphi }}_{0}}$ corresponded to a two-dimensional (2D) torus of Equation (12).

Topological changes in the ω-limit sets of the extended system Equation (12) could thus be understood via bifurcations of the discrete map $P_{{\bar{\varphi }}_{0}}$. Specifically, the bifurcation analysis we performed for autonomous system (6) also applied to the Poincaré map $P_{{\bar{\varphi }}_{0}}$. Hopf bifurcations undergone in autonomous system (6) which gave rise to limit cycles in 3D phase space corresponded to *Neimark-Sacker bifurcations* of $P_{{\bar{\varphi }}_{0}}$ which gave birth to a 2D torus in the extended space. The torus oscillation thus had two periodic components: one (the toroidal direction) driven extrinsically by the frequency of sniffs and the other (the poloidal direction) governed by the intrinsic network dynamics. Therefore, the Neimark-Sacker bifurcations provided an analogous bifurcation mechanism for non-autonomous system (10) as the Hopf bifurcations did for autonomous system (6).

## Results

### Reduced Network Model Generates Complex Dynamics

To understand the functional role of top-down projections onto inhibitory neurons, we built a three-node network model (Figure 1A, see Methods) that recapitulated a circuit architecture identified both structurally (Padmanabhan et al., 2019) and functionally (Boyd et al., 2012; Markopoulos et al., 2012) across a number of brain areas. For different stimuli μ, the network exhibited a variety of dynamics (Figure 1B). For instance, when the stimulus was small, the firing rates *r*_*i*_, *i* = 1, 2, 3 had a fast-transient increase followed by damping oscillations that converged to a stationary state (Figure 1B, left). A sufficiently large stimulus μ elevated the firing rates to near saturation, where they then remained at the upper bound of the non-linear sigmoid function throughout the duration of the stimulus (Figure 1B, right). For small or large stimuli, the network responses converged to a constant firing rate after transient dynamics. By contrast, for medium values of μ, more complex firing rate dynamics emerged, including oscillations (Figure 1B, middle). To visualize the collective behaviors of M, G, and P populations to these different stimuli, we turned to a three dimensional dynamical system representation of the model where the time evolution of the firing rates (i.e., *state variables*) was a *trajectory* (or an *orbit*) in the *phase space* (*r*_1_, *r*_2_, *r*_3_) and the tangent vector defining the velocity of each point along a trajectory was given by the *vector field* $f={\left(f_{1},f_{2},f_{3}\right)}^{T}$ (see Methods) of Equation (1). The firing rates over time in Figure 1B thus corresponded to trajectories in Figure 1D starting from the origin O (where all three populations were silent). For small stimuli, the trajectory made an excursion before spiraling into an *equilibrium* indicated by the solid dot (Figure 1D, orange). Similarly, when the stimulus μ was large, the trajectory again settled into an equilibrium, but one that was translated within the phase space to the top-right corner (Figure 1D, black). Finally, for medium stimuli, the time-varying oscillation of firing rates manifested as a *periodic orbit* (or a *limit cycle*) in the 3D phase space (Figure 1D, brown). By convention, we defined the steady-state dynamics as the ω*-limit set* of the system.

### Top-Down Weight Reshapes Network Dynamics and Modulates Neural Oscillations

Next, to explore how top-down down projections onto the inhibitory granule cell population (G) shaped the dynamics of the network, we studied the effects of changes in the connection weight *w*_23_ on firing rate dynamics. First, we varied the top-down weight *w*_23_ (Figure 2A, top) from the piriform population (P) to the inhibitory granule cell population (G) and studied the effect of these changes on the firing rate dynamics of the network. For a fixed stimulus (μ = 1.5) the dynamics of the firing rates *r*_*i*_ (*t*), *i* = 1, 2, 3 were sensitive to different values of *w*_23_ (Figure 2A, bottom). When the top down weight was small (*w*_23_ = 4), firing rates approached the equilibrium exponentially (Figure 2A, bottom and Figure 2B, left, black traces). Conversely, when the top-down weight was large (*w*_23_ = 10.5), the firing rate of excitatory cells (*r*_1_) increased initially, but was suppressed as inhibition reduced the activity, until the firing rates ultimately settled to an equilibrium (Figure 2A, top and Figure 2B, left, light magenta traces). When the magnitude of top-down weight was changed to an intermediate value (*w*_23_ = 5.5), the same stimulus generated oscillatory activity in the network, with the steady-state dynamics transitioning to a periodic orbit (a limit cycle). Changing the weight of top-down projections onto the local inhibitory population for a single stimulus produced the same diversity of firing rate dynamics that occurred from changes in the stimulus. Furthermore, for a given top-down weight (*w*_23_), the effects on the network dynamics stimulus was unique to that stimulus (Figure 2B, right vs. left).

In regimes where specific weights of top-down weight generated sustained oscillatory activity for a given stimulus μ, we characterized the frequency and amplitude of these oscillations (Figure 2B, left *w*_23_ = 5.5, right *w*_23_ = 10.5) as changes in both have been tied to circuit function and behavior (Buzsaki and Draguhn, 2004; Kay et al., 2009). For a given stimulus, oscillations emerged between two values of *w*_23_, with the frequency of the oscillation varying monotonically (Figure 2C1). By contrast, while the amplitudes of the oscillations started from zero at the two critical values of *w*_23_, they reached a maximum in between (Figure 2D1). The control of both the frequency and amplitude via changes in *w*_23_ occurred across an array of weights (*w*_31_) associated with the feedforward drive from the mitral/tufted population (M) to the piriform population (P) (Figures 2C2,D2). Furthermore, the magnitude of synaptic weights from mitral/tufted cells to piriform cortical neurons established the dynamic range within which changes in top-down weights (*w*_23_) influenced the frequencies (Figure 2C3, 0 − 45Hz) and amplitudes (Figure 2D3, 0 − 1 A.U.) of network oscillations, spanning frequencies in the alpha, beta and gamma bands.

### Top-Down Weight Contributes to Pattern Separation

As changing the top-down weight onto inhibitory neurons could generate complex activity patterns we next asked what computations could be performed by this control. For example, both behavioral and neurophysiological measures show that as the representations of two stimuli by neuronal circuits become different, distinguishing between them becomes easier (Friedrich and Laurent, 2001; Leutgeb et al., 2007; Yassa and Stark, 2011) Control of inhibition, via top-down centrifugal projections, may be one way that such stimulus discrimination is implemented by the circuit.

To test this hypothesis, we presented our network with a pair of stimuli, denoted by μ_1_ and μ_2_ (corresponding to stimuli arranged along a one-dimensional axis) and studied how control of inhibition altered the representations of the two stimuli (Figure 3A). Conceptually, these two stimuli could be two different concentrations of an odor or two odors that share a similar physiochemical feature (two odors with different carbon chain lengths). For a set of stimuli μ_*i*_, *i* = 1, 2, we defined the steady-state *representation* of network activity as the ω-limit set Ω_*i*_, *i* = 1, 2. The distance between the two stimuli μ_1_ and μ_2_ in the stimulus space was defined as Δμ, and the resultant distance in the firing rate phase space between the two ω-limit sets (Ω_1_ and Ω_2_) we defined as a metric *d* (see Methods). The smaller the Δμ, the more similar the two stimuli were. We hypothesized that changes in the weight of feedback onto the inhibitory neuron population (*w*_23_) could increase the value of *d*, making the representations of those stimuli more distinct (Figure 3A).

In a representative example where μ_1_ = 1.0 and μ_2_ = 1.1, when the top-down weight was low (*w*_23_ = 3), the representations of the two stimuli were close (Figures 3B,C, left). As *w*_23_ was increased, the representations of the two stimuli were pushed apart making them more separable (*w*_23_ = 3.8, Figures 3B,C). Interestingly, as *w*_23_ was increased further (*w*_23_ = 10), the representations of the two stimuli became close to one another again (Figures 3B,C, right). As representations could be either oscillations in the state space, or equilibria, we compared how the distances of these representations changed across different measures (see methods). Interestingly, although the absolute values given by the Euclidean distance *d*^*E*^ (Figure 3C) and the spectrum distance *d*^*S*^ (Figure 3B) were different, they both occurred at the same feedback weight (Figure 3D). We visualized the distance landscapes defined by *d*^*E*^ and *d*^*S*^ over all combinations of μ_1_ and Δμ as a function of a change in the weight of the top-down weight (Figures 4A,B). Irrespective of which distance definition was exploited for measurement, we found an optimal value of $w_{23}^{\max}$ that maximized the distance between the two resultant representations for any given pair of stimuli.

A landscape of the optimal $w_{23}^{\max}$ across all pairs of stimuli (μ_1_, μ_2_) was shown in Figure 4C. Thus, changing the weight of top-down projections onto the inhibitory neuron population could be used to facilitate stimulus separation dynamically. To understand why, we examined the effect that varying the top-down weight had on the firing rate responses of both local excitatory mitral/tufted cell and inhibitory granule cell populations (*r*_1_ and *r*_2_). For a given stimulus, an increase in *w*_23_ led to a monotonic decrease in *r*_1_, suggesting persistent suppression onto the local M population. By contrast, the response of the local G population *r*_2_ was elevated first with increasing *w*_23_ until reaching the maximum $r_{2}^{\max}$, after which *r*_2_ dropped significantly (Figure 4D). Across different stimuli μ, the shape of the firing rate *r*_2_ as a function of *w*_23_ remained the same but shifted vertically. To determine if these differences in the firing rate of inhibitory neurons (*r*_2_) were related to the values of top-down weights that maximally separated the distance between two stimulus representations, we plotted the $w_{23}^{\max}$ (abscissa) obtained from the landscape in Figure 4C vs. the midpoint $w_{23}^{mid}$ (ordinate) between the $r_{2}^{\max}$ of the same stimulus pair (inset of Figure 4E). The response *r*_2_ to one stimulus on the left of the midpoint dropped significantly, while the response to a similar stimulus on the right still had a high inhibitory firing rate. The optimal $w_{23}^{\max}$ was correlated to $w_{23}^{mid}$ (*R*^2^ = 0.98), the value at which inhibitory neuron activity from one stimulus was suppressed while activity from the other similar stimulus remained persistently high. Consequently, stimulus separation arose from the differential sensitivity of inhibitory neurons to the balance between top-down feedback and recurrent inhibition; an imbalance occurred between the top-down feedback and the recurrent inhibition for one stimulus while that balance was preserved for the second stimulus.

### Top-Down Weight Contributes to Oscillation Synchrony

**Figure 5:**
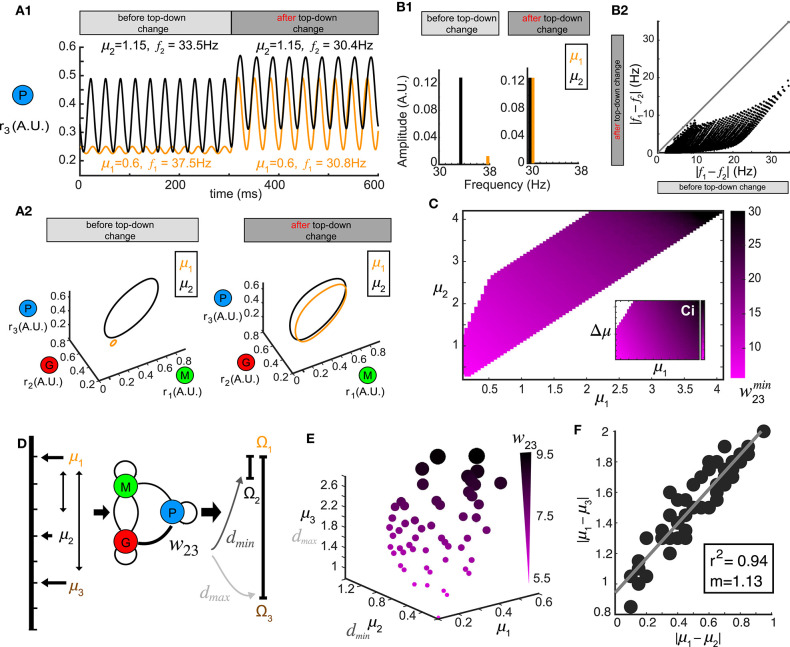
Oscillation synchrony via top-down control. **(A)** Oscillatory responses to two example stimuli μ_1_ = 1.15 and μ_2_ = 0.6 become synchronized promptly after changing the top-down input *w*_23_. **(A1)** Time series of *r*_3_ (*t*) before and after changing the top-down input. **(A2)** Limit cycles corresponding to the oscillatory responses in **(A1)** are plotted in phase space (transitions not shown). **(B)** Changing *w*_23_ can make both the frequency and amplitude of two oscillations closer to each other. **(B1)** Frequency and amplitude components of the two oscillations shown in **(A)**. **(B2)** Changing top-down input reduces the frequency differences of responses to two distinct stimuli, effectively using frequency to synchronize the representations in the phase space. **(C)** The matrix $w_{23}^{\text{min}}$ which minimizes the distance *d* between oscillatory responses to all combinations of stimuli μ_1_ and μ_2_. **(D)** Schematic diagram illustrating that the same value of *w*_23_ which minimizes the distance between oscillations responding to stimuli μ_1_ and μ_2_ can maximize the distance between responses to stimuli μ_1_ and μ_3_. **(E)** Scatter plot in μ_1_- μ_2_- μ_3_ space where each sphere denotes a top-down input *w*_23_ as illustrated in **(D)** and is coded by color and size. **(F)** Correlation between the differences of those stimuli of which the response distances are simultaneously minimized and maximized. Inset: correlation coefficient and the slope of linear regression.

Stimulus-evoked oscillations also appeared in our model, and were modulated by the top-down weights (Figure 2) covering a wide range of frequency and amplitude. This suggested that oscillatory responses to different stimuli could be synchronized by tuning *w*_23_. To explore this, we first examined the oscillations in the firing rate generated by two different stimuli μ_1_ and μ_2_. At a given value of top-down weight (*w*_23_ = 12.0), one stimulus (μ_1_ = 1.15) generated oscillations (*f*_1_ = 33.5 Hz, Figure 5A1, before) in the piriform population's firing rate that were different in both frequency and amplitude from the oscillations (*f*_2_ = 37.Hz, Figure 5A1, before) in response to a second stimulus (μ_2_ = 0.6). However, a change in the top-down weight (*w*_23_ = 8), resulted in firing rate oscillations becoming more similar for the same two stimuli (Figure 5A1, after, *f*_1_ = 30.4 Hz, *f*_2_ = 30.8 Hz). This increase in the firing rate synchrony was also apparent when visualized in the 3D phase space (Figure 5A2). To quantify the synchrony between the oscillations responses to μ_1_ and μ_2_, we calculated the spectrum distance *d*^*S*^ (see Methods) between the network representations for the two stimuli before and after changes in top-down weight (Figure 5B1). Changes in the feedback to inhibitory neurons *w*_23_ synchronized activity in the network stimuli (Figure 5B2), and while the effect was greatest when stimuli were similar, we found examples for stimuli that were initially as far apart as 20 Hz. As with stimulus discrimination, a systematic relationship emerged corresponding to the optimal top-down weight $w_{23}^{\min}$ across combinations of stimuli (μ_1_ vs. μ_2_) that was most effective at generating synchronous oscillations (Figure 5C).

Although we have thus far treated stimulus discrimination and synchrony separately, neural circuits perform both operations simultaneously, bringing the network representation of one stimulus closer to another, while simultaneously pushing the representation of that stimulus farther from a third. We therefore tested if a single change in the top-down weight *w*_23_ accomplish both of these operations; minimize the distance between the responses to one pair of stimuli (μ_1_ vs. μ_2_) while also maximize the response distance to another other pair of stimuli (μ_1_ vs. μ_3_, Figure 5D). To do this, we generated a 3D scatter plot of values of *w*_23_ that were optimal for synchrony between oscillations generated by stimulus μ_1_ and μ_2_ (Figure 5C) and also produced a maximum separation between the representations of stimulus μ_1_ and μ_3_ (Figure 5E). The values of top-down weight *w*_23_ for each point that fulfilled these diametrically distinct functions were coded by color and size (Figure 5E). We found that the top-down weight corresponding to both operations scaled with the stimuli, such that when μ_1_, μ_2_ and μ_3_ were small, the top-down weight was also small, but as the three stimuli increased in magnitude, the top-down weight needed to synchronize one pair and separate the other pair also increased. Finally, we found a strong correlation between the values of stimulus differences: |μ_1_ − μ_2_| and |μ_1_ − μ_3_| (Figure 5F) at which an *w*_23_ weight was optimal for stimulus separation and oscillatory synchrony.

### Generalization to Oscillatory Stimulus Driven by Sniffs

**Figure 6:**
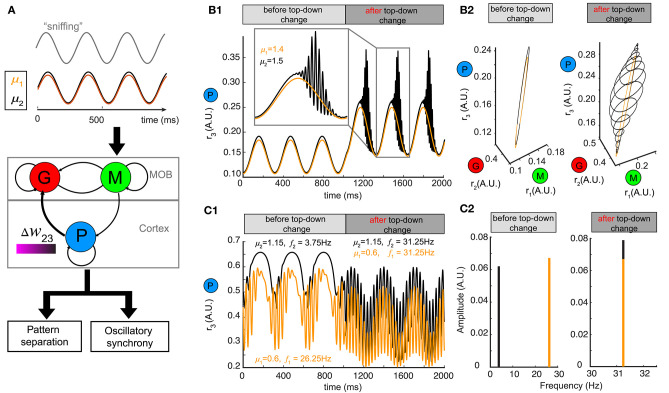
Pattern separation and oscillation synchrony for sniff-modulated oscillatory stimulus. **(A)** Schematic diagram illustrating that varying the top-down weight of the network model (middle) can accomplish both pattern separation and oscillation synchrony (bottom) for a pair of oscillatory stimuli μ_1_ and μ_2_ modulated by sniffs (top). **(B)** Pattern separation for two oscillatory stimuli with closely related amplitudes: μ_1_ = 1.4 and μ_2_ = 1.5. **(B1)** Firing rate of the piriform population *r*_3_ (*t*) before and after changing the top-down weight: before, *w*_23_ = 26; after, *w*_23_ = 21. **(B2)** The limit cycles in the phase space corresponding to the firing rate in **(B1)**. **(C)** Oscillation synchrony for two oscillatory stimuli with distinct amplitudes: μ_1_ = 1.15 and μ_2_ = 0.6. **(C1)** Changing the top-down weight from *w*_23_ = 6.1 to *w*_23_ = 9.2 synchronizes the intrinsic oscillations of the firing rate of the piriform population *r*_3_ (*t*). **(C2)** Frequency and amplitude components of the two intrinsic oscillations shown in **(C1)**.

Although we used a constant stimulus μ to represent the average input to mitral/tufted cells, in mammals sniffing brings odors into the nasal epithelium in a periodic fashion (Wachowiak, 2011). Sniff cycles carry different amounts of information about odor identity and concentration (Miura et al., 2012) and a single sniff cycle is sufficient for animals to discriminate accurately between two odors (Uchida and Mainen, 2003; Wesson et al., 2008). To explore how changing top-down weights can reshape network responses to oscillatory stimuli, we modeled our stimulus μ as a sinusoidal function $\tilde{\mu }\left(t\right)=\mu \cos\left(\omega_{s}t+\varphi_{0}\right)$, where the different odors had different amplitudes μ, the sniffing frequency ω_*s*_ was set to ~4 Hz and φ_0_ characterized the initial phase of sniffing (Carey and Wachowiak, 2011; Shusterman et al., 2011) (Figure 6A).

For a pair of oscillatory stimuli $\tilde{\mu }\left(t\right)$ with two similar amplitudes μ_1_ and μ_2_, the firing rate responses were also similar for the piriform population (P) (Figure 6B1, before top-down change) and the entire network in the phase space (Figure 6B2, left). If we changed the top-down weight *w*_23_, as we had done for a fixed stimulus, both the piriform population firing (Figure 6B1, after top-down change) and the network representations became more distinct (Figure 6B2, right). The conch-shaped limit cycle in Figure 6B2 (right) arose from oscillations occurring at two different time scales (see methods): a slower oscillation on the time scale of sniff cycles and a faster oscillation governed by the intrinsic dynamics of the network. As a consequence, the same pattern separation achieved by changing top-down weight for constant stimuli could also be accomplished for oscillatory stimuli.

To explore if network representations of oscillatory stimuli could be made synchronous by changing top-down weights, we presented two stimuli with distinct amplitudes (μ_1_, μ_2_) to the network (Figure 6A). Following a change in the top-down weight *w*_23_, the network representations became synchronous (Figure 6C1), with the oscillations of firing rates occurring at the same frequency (Figure 6C2, right). Importantly, these high frequency oscillations occurred at the gamma band, and rode on top of the slower oscillations corresponding to sniff cycles, further revealing the computational decoupling of sniffing and inhibitory dynamics across two different time scales. Taken together, the mechanisms giving rise to both pattern separation and oscillatory synchrony were general to constant and oscillatory inputs.

### Bifurcation Mechanism for Top-Down Control of Inhibition

Finally, to understand mathematically how such operations emerged from changes in the top-down weight to inhibition, we studied the structure of the transitions in network firing rates dynamics (Figure 2A). These transitions were associated with qualitative or topological changes in the ω-limit sets of the system, indicative of the occurrence of bifurcations in the system.

**Figure 7:**
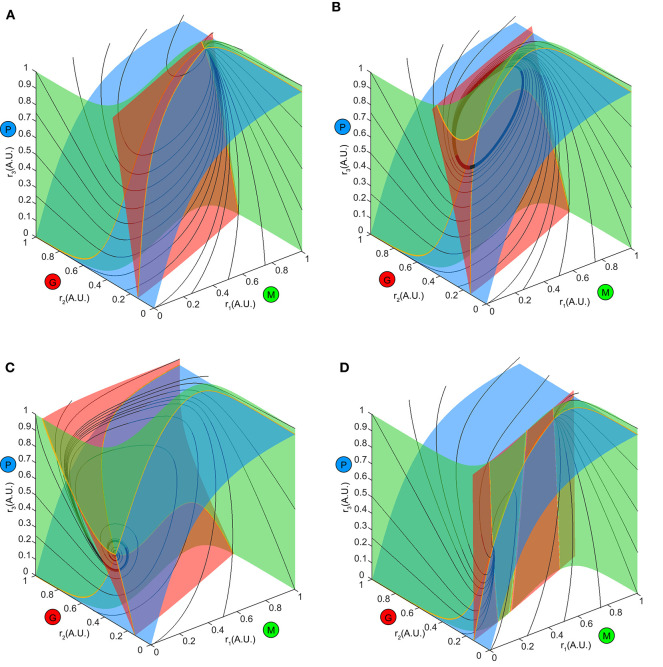
Global phase structure changes with the top-down input. **(A–C)** Global phase structures showing the nullclines (yellow thick curves), nullplanes (transparent surfaces with the same color code as M, G, P population) and several representative trajectories (black thin curves) for the same stimulus μ = 1.5 and three different values of *w*_23_. A, *w*_23_ = 4; B, *w*_23_ = 6; C, *w*_23_ = 15. Varying the top-down input tilts the nullplanes, thus changing the position of the equilibrium as well as its stability. **(D)** An example phase structure where three equilibria were present simultaneously (two stable and one unstable), corresponding one of the blue regions of the manifold in Figure 8A.

**Figure 8:**
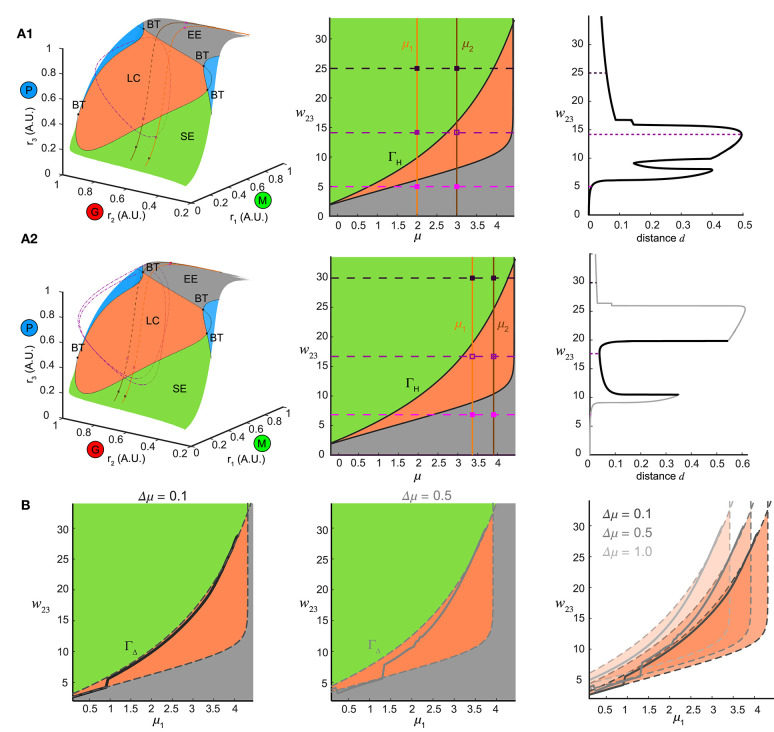
Bifurcation mechanism of top-down control to support both pattern separation and oscillation synchrony. **(A)** Illustration of the bifurcation mechanism and the transition boundary of dynamics in phase space and parameter space. **(A1)** Pattern separation. Left: the equilibrium manifold in the firing rate phase space divided by the separatrix emitting from multiple codimension-2 bifurcation points: BT (Bogdanov–Takens bifurcation) into several regions. Region-LC: each equilibrium was unstable and had exactly one corresponding stable limit cycle (dot dashed cycle) arising from a Hopf bifurcation. Region-SE: each equilibrium was stable all trajectories spiraled into it. Region-EE: each equilibrium was stable all trajectories approached it exponentially. Blue regions: regions where multiple equilibria coexisted. For two example stimuli μ_1_ = 2.0 and μ_2_ = 3.0 given in the middle of **(A1)**, two paths of equilibria were induced on the equilibrium manifold and traversed across different regions as changing top-down input *w*_23_. Middle: different regions on the equilibrium manifold corresponded to different regimes in the parameter space of μ and *w*_23_ in the same color scheme [parameters for blue regions in Left were largely beyond the range thus not shown]. The transition boundary Γ_H_ specified the pair (μ, *w*_23_) at which the network underwent a Hopf bifurcation and corresponded to the separatrix enclosing the region-LC in Left. Two given stimuli were denoted by two vertical lines and three example values of *w*_23_ corresponded to three horizontal dashed lines, giving rise to a pair of junctions for each. These junctions were also plotted as squares in the left of **(A1)** denoting the corresponding ω-limit sets in the same color (solid square: stable equilibrium; empty square: unstable equilibrium). Right: the distance between the ω-limit sets to represent the two given stimuli. The maximal distance was achieved when the two junctions were on opposite sides of Γ_H_. **(A2)** Same as **(A1)** but for oscillation synchrony occurring when the two junctions were both inside Γ_H_. **(B)** Comparisons between the translated transition boundary Γ_Δ_ (dashed curve) depending on μ (left: Δμ = 0.1, middle: Δμ = 0.5) and the sliced section of the $w_{23}^{\max}$ at the same Γμ (solid curve). Right: a series of translated Γ_Δ_ for three representative values of Γμ.

To explore this further, we first examined the how the ω-limit sets of the system receiving constant stimuli changed with different top-down weights. An equilibrium corresponding to constant firing rates in the network arose from the intersection of three nullclines (Figure 7, yellow thick lines), each resulting from pairwise intersections of three “nullplanes” that characterized the geometric surface on which the firing rate derivatives of one node equaled to zero (Figure 7, transparent surfaces). Global phase structures for two representative values of top-down weights (Figure 7A: ω_23_ = 4, Figure 7C: ω_23_ = 15) illustrated how these equilibria varied within the firing rate phase space. In these two examples, both equilibria were stable and attractive, with all nearby trajectories (Figures 7A,C, black thin lines) moving toward them. This was, however, not true for all values of *w*_23_. At some critical values of *w*_23_, the equilibrium lost stability, and a small-amplitude limit cycle branched from that unstable equilibrium, resulting in the oscillations observed in the dynamics (Figure 7B). This transition signified a *Hopf* bifurcation of the system (see Methods), which arose when the top-down weight *w*_23_ was within a specific regime. Therefore, across all combinations of external stimuli μ and top-down weight *w*_23_, we obtained a smooth manifold in the phase space (Figure 8A, left), corresponding to a family of ω-limit sets on which the network dynamics settled from any set of initial conditions. Sustained oscillations corresponded to the red region-LC (LC: limit cycle) where each equilibrium (unstable) was paired with exactly one limit cycle born simultaneously via a Hopf bifurcation (the purple empty square vs. the dot-dashed curve). Constant firing rates corresponded to the gray region-EE (EE: exponential equilibrium) and green region-SE (SE: spiraling equilibrium), where the equilibria were stable, approached either exponentially (region-EE) or via damping-oscillations (region-SE). Finally, the two blue regions on the manifold were bounded by saddle-node bifurcations near Bogdanov–Takens (BT), an example global phase structure of which was shown in Figure 7D. The equilibrium manifold thus defined the entire family of network representations for all possible combinations of stimuli μ and top-down weigh t*w*_23_.

Within the manifold of the stimulus μ and top-down weight *w*_23_, we identified a transition boundary Γ_H_ (black solid curves, Figure 8A, middle) corresponding to the separatrix enclosing the region-LC. Γ_H_ specified the parameter pairs (μ, *w*_23_) at which a Hopf bifurcation occurred, thereby dividing the parameter space into regimes with different dynamics (same color coded as Figure 8A1, left). For a given pair of stimuli (for example, μ_1_ = 2.0, μ_2_ = 3.0), changing *w*_23_ corresponded to shifting the horizontal dashed line vertically (three representatives were shown in Figure 8A1, middle), thereby shifting the junctions with the two stimuli (vertical solid lines, Figure 8A1, middle) across different regimes in the parameter space. In the firing rate phase space (Figure 8A, left), these changes in *w*_23_ for one stimulus moved the equilibrium through different regions of the manifold: EE-LC-SE, while for another stimulus, a parallel curve on the manifold could also be traced. When the two junctions in Figure 8A1 (middle) were on different sides of the transition boundary, with one equilibrium in region-LC and the other in region-SE (Figure 8A1, left), the two network representations became topologically different from each other; the former a limit cycle, and the latter an equilibrium point. Thus for a combination of stimulus pairs, the optimal ${w_{23}}^{\max}$ for pattern separation was then achieved when the ω-limit sets were on different sides of transition boundary (Figure 8A1, right).

Furthermore, when the junction of feedback weight and stimulus pair were both inside the transition boundary (Figure 8A2, middle) two limit cycles emerged (one for each stimulus, for example, μ_1_ = 3.45, μ_2_ = 3.95, Figure 8A2) synchronize the network representations. Changes in top-down weight moved the junctions for pairs of stimuli within the parameter space, revealing a shared mechanism supported both stimulus separation and oscillation synchrony, depending on the relative positions of the junctions with respect to the transition boundary.

Finally we determined if the transition boundary identified via analysis of the dynamical system corresponded to the $w_{23}^{\max}$ matrix found in Figure 4Ci. To do this, we considered a set of initial stimuli μ_1_, and a set of distances to a second set of stimuli Δμ, wherein each value was an array that defined a set of stimulus pairs {(μ_1_, μ_1_ + Δμ)| μ_1_ ∈ [0, 4]}. For a given Δμ > 0, distinguishing the pair (μ_1_, μ_1_ + Δμ) was the same as distinguishing (μ_1_ + μ, Δμ_1_) in terms of pattern separation. In this framework two different distances, for instance, Δμ = 0.1 or μ = 0.5 (Figure 8B, left and middle), the set of stimulus pairs had a unique transition boundary Γ_Δ_ (Figure 8B, right). The section of the $w_{23}^{\max}$ matrix in Figure 4 Ci for the set of stimulus pairs {(μ_1_, μ_1_ + Δμ)| μ_1_ ∈ [0, 4], Δ*μ is given*} was correlated with the transition boundary Γ_Δ_ of the same value μ (Figure 8B). For small Δμ = 0.1, the slice of the $w_{23}^{\max}$ followed closely with the transition boundary Γ_Δ_ (Figure 8B, left). As the stimulus difference increased (Figure 8B, middle), $w_{23}^{\max}$ deviated from the boundary Γ_Δ_. Larger Δμ's increased the bifurcation lag between two stimuli such that the stimulus that caused a bifurcation first had more parameter space to develop before the bifurcation of the other stimulus. Conversely, stimulus discrimination was harder as μ decreased because the range of top-down weights *w*_23_ that separate two stimuli shrank significantly around a close vicinity of the transition boundary. Thus, subtle adjustments of top-down weight around the transition boundary were required to separate similar stimuli from each other. The same analysis could also be performed for the non-autonomous system receiving oscillatory stimuli $\tilde{\mu }\left(t\right)$ by investigating bifurcations of fixed points of the constructed Poincaré map on a given cross section (see Methods) with the same computational mechanism arising via the discrete version of Hopf bifurcation, i.e., a Neimark-Sacker bifurcation (Kuznetsov, 2013). Taken together, these results provide a bridge linking the mechanisms that give rise to the dynamics of the neural circuit with the computations performed by the circuit.

## Discussion

Using a three-node model, which included top-down projections from piriform cortical cells onto inhibitory granule cells in the main olfactory bulb, we identified a network capable of complex dynamic behaviors, ranging from an attractor to stable oscillations across a range of frequencies and amplitudes. By changing the weight of these top-down projections, the network could either facilitate pattern separation between two similar stimuli, or synchronize the oscillatory activity produced by two different stimuli. A bifurcation analysis of the dynamical system revealed that both mechanisms emerged from the transition boundary of Hopf bifurcations which branched from co-dimensional two bifurcation points (i.e., the Bogdanov-Takens bifurcation). Furthermore, these computations could be accomplished even when the stimuli were periodic, fluctuating at the frequency of sniffing (Neimark-Sacker bifurcation), suggesting that these findings are a general feature of this network. Our results provide both a mathematical framework for how top-down control of inhibition shapes the dynamics of a network, and a link between such dynamics and the computations that neural circuits can perform.

An important point to consider is how changes in top-down weights may be instantiated biologically? This point depends on the timescale of weight changes. On short time scales, changes in inhibitory drive to granule cells can facilitate olfactory discrimination (Abraham et al., 2010; Nunes and Kuner, 2015) and generate synchronous oscillatory activity among mitral cells in the bulb (Galan et al., 2006). Neuromodulators such as serotonin (Petzold et al., 2009; Kapoor et al., 2016) can act on fast sub second time scales to support both oscillatory synchrony and stimulus discrimination, providing one biological mechanism by which weights can be changed dynamically. By contrast, long-term changes in the bulb may be instantiated by classis synaptic plasticity mechanisms such as LTP (Cauthron and Stripling, 2014), or via the remodeling of synaptic connectivity (Arenkiel et al., 2011; Deshpande et al., 2013), for instance due to adult neurogenesis (Lledo et al., 2006). In these examples, the changes in feedback weight likely reflect slow alterations in network structure that result in stable changes in neural representations, possibly corresponding to learning.

While the biological mechanisms by which the top-down synaptic weights change onto inhibitory neurons may be diverse depending on timescale, we find that such alterations give rise to functionally equivalent changes supporting an array of computations. For instance, changes in the top-down weight would render two stimuli more distinct at the level of firing rates in the population, a process referred to as pattern separation (Cayco-Gajic and Silver, 2019) or decorrelation (Friedrich and Laurent, 2001). Our model predicts that pattern separation arises from the non-monotonic change in firing in granule cells (at the balance between op-down excitation and recurrent inhibition). The top-down weight onto inhibitory neurons sets a gate, allowing some stimuli to cross a threshold of recurrent inhibition, while others do not.

In parallel, changing top-down weights onto inhibitory neurons can increase the synchrony between two stimuli that were initially asynchronous. A number of experimental and theoretical studies have explored the privileged role that inhibitory interneurons play in generating gamma oscillations (Whittington et al., 1995; Hasenstaub et al., 2005; Cardin et al., 2009; Sohal et al., 2009; Tiesinga and Sejnowski, 2009). Among these, the two most common models are when gamma arises from reciprocal coupling between pyramidal cells and inhibitory interneurons (PING), and recurrent connections among inhibitory interneurons (ING) (Whittington et al., 2000; Tiesinga and Sejnowski, 2009). In both, oscillatory activity arises from the structure of local connectivity. In our work, we identified another motif by which gamma oscillations can arise—**T**op-down control of **I**nhibitory **N**euron **G**amma (TING). Local excitatory mitral and tufted cells broadcast activity patterns to a pyramidal/semilunar cell population in piriform cortex, that then synapses back onto inhibitory granule cells.

Studies on dynamics of local excitatory and inhibitory neurons in the olfactory system both experimentally and mathematically are extensive (Wilson and Cowan, 1972; Ermentrout and Kopell, 1990; Kay et al., 2009; Li and Cleland, 2017). To these models we add a description of how an external (in this case, top-down input from piriform cortex) source controlling the inhibitory neuron population can influence dynamics. In studying the dynamical system defined by this network, we found that the bifurcations largely result from the singularity (linearized Jacobian matrix is non-hyperbolic) inherently embedded in the system itself. Thus, although the exact parameter values (defined by the weights of connections) influence when the dynamics of the network undergoes a bifurcation, the types of bifurcations that arise are determined by the normal form of the system (Guckenheimer and Holmes, 2013; Kuznetsov, 2013); revealing that the behaviors observed in this three node population are a fundamental feature of the network architecture. For Wilson-Cowan equations we used, the Bogdanov–Takens bifurcation is the inherent codimension-2 singularity (Cowan et al., 2016), meaning that the diversity of dynamics exists for a broad range of parameter settings, and that the unfolding of these dynamics can be implemented by modulating the top-down connection weight. Our model address this in the context of olfaction (Oswald and Urban, 2012a), but it may be applicable to a number of other sensory systems that share a similar architecture. For instance, the axonal projections from the cingulate of frontal cortex to GABAergic inhibitory neurons in V1 of the mouse visual system are organized (Zhang et al., 2014), and may therefore serve an analogous function as piriform projections to granule cells. Consequently, we identified a generalized principle by which control of inhibition via top-down weights can support a number of computations essential for neural circuit function.

Finally, we found that the firing rate representations of mitral/tufted cells, granule cells, and piriform neurons resided within distinct domains on a manifold defined by the stimulus and the weight of feedback. These domains corresponded to transitions in the dynamics of the system. Changes in the top-down weights moved a transition boundary that delineating these domains across different stimuli. When two stimuli were on opposites sides of this transition boundary, their dynamics operated under two different regimes, and their representations were pushed further apart. By contrast, when the stimuli were both on the same side of the transition boundary, within regimes corresponding to similar dynamics, their activity became more synchronous; effectively binding those stimuli together. Changes in top-down weight were therefore changes in the location of the transition boundary that could either marshal the representations of two stimuli together or push them apart. In conclusion, we identified a model that links the dynamics of neural systems with the computations they are hypothesized to perform and may be used as a generalized framework to study the diverse effects of feedback onto inhibitory populations.

## Data Availability Statement

The raw data supporting the conclusions of this article will be made available by the authors, without undue reservation.

## Author Contributions

KP conceived and supervised the project. ZC performed all the experiments and analysis. ZC and KP made the figures and wrote the manuscript. All authors contributed to the article and approved the submitted version.

## Conflict of Interest

The authors declare that the research was conducted in the absence of any commercial or financial relationships that could be construed as a potential conflict of interest.
